# Using simulation for training and to change protocol during the outbreak of severe acute respiratory syndrome

**DOI:** 10.1186/cc3916

**Published:** 2005-11-24

**Authors:** Simon D Abrahamson, Sonya Canzian, Fabrice Brunet

**Affiliations:** 1 Assistant Professor of Anesthesia, Department of Anesthesia and Division of Critical Care, University of Toronto, St. Michael's Hospital, 30 Bond Street, Toronto, M5W 1W8, Canada; 2 Clinical Leader Manager, Trauma and Neurosurgery Intensive Care Unit, St. Michael's Hospital, 30 Bond Street, Toronto, M5W 1W8, Canada; 3 Professor of Medicine, Department of Medicine and Division of Critical Care, University of Toronto, St. Michael's Hospital, 30 Bond Street, Toronto, M5W 1W8, Canada

## Abstract

**Introduction:**

During the 2003 severe acute respiratory syndrome (SARS) crisis, we proposed and tested a new protocol for cardiac arrest in a patient with SARS. The protocol was rapidly and effectively instituted by teamwork training using high-fidelity simulation.

**Methods:**

Phase 1 was a curriculum design of a SARS-specific cardiac arrest protocol in three steps: planning the new protocol, repeated simulations of this protocol in a classroom, and a subsequent simulation of a cardiac arrest on a hospital ward. Phase 2 was the training of 275 healthcare workers (HCWs) using the new protocol. Training involved a seminar, practice in wearing the mandatory personal protection system (PPS), and cardiac arrest simulations with subsequent debriefing.

**Results:**

Simulation provided insights that had not been considered in earlier phases of development. For example, a single person can don a PPS worn for the SARS patient in 1 1/2 minutes. However, when multiple members of a cardiac arrest team were dressing simultaneously, the time to don the PPS increased to between 3 1/2 and 5 1/2 minutes. Errors in infection control as well as in medical management of advanced cardiac life support (ACLS) were corrected.

**Conclusion:**

During the SARS crisis, real-time use of a high-fidelity simulator allowed the training of 275 HCWs in 2 weeks, with debriefing and error management. HCWs were required to manage the SARS cardiac arrest wearing unfamiliar equipment and following a modified ACLS protocol. The insight gained from this experience will be valuable for future infectious disease challenges in critical care.

## Introduction

Severe acute respiratory syndrome (SARS) is a newly identified atypical pneumonia that can be life threatening. Attention was drawn to the disease in February 2003 when a physician and subsequently 12 other hotel guests staying in a hotel in Hong Kong became ill [1]. One of these hotel guests returned to Toronto, Canada, died on 5 March 2003, and became the index case for Toronto. The *Morbidity and Mortality Weekly Report *published a description of the SARS outbreak on 21 March 2003 [2]. The SARS virus seemed to be highly contagious in the hospital setting. A case report suggested that intubation of patients produced a high risk for transmission of SARS to healthcare workers (HCWs) [3].

SARS created a crisis in healthcare in Toronto. The lack of literature, uncertainty about treatment, and fear of the disease caused great concern among HCWs. In late April 2003, our Critical Care Department was asked to urgently develop and implement a protocol for the management of cardiac arrest in the SARS patients. At the time there were directives from the Ontario provincial government mandating the use of a personal protection system (PPS) during the intubation of SARS patients [4]. A PPS was defined as 'an apparatus consisting of head, face and neck protection with or without enclosed body protection'. An example of a PPS cited in the directive was the Stryker T4 system (Stryker^® ^Instruments, Kalamazoo, MI, USA).

The cardiac arrest scenario was of great concern because care had to be delivered immediately. We knew from previous simulation experience that a HCW required 1 1/2 minutes to dress in the Stryker T4 [5]. Hence, application of a PPS would increase the time before resuscitation could begin. We needed to develop a protocol that ensured HCW safety as well as timely patient care.

We used simulation to perfect the protocol as well as to train the cardiac arrest team. Simulation has been used to improve individual and team performances [6-9]. It has also been used as an evaluative tool [10,11]. We used the simulated cardiac arrest scenarios to provide an opportunity for deliberate practice, an important concept in effective learning [12]. The rationale for this approach was that simulation improved the retention of advanced cardiac life support (ACLS) guidelines in comparison with textbook review [13].

## Materials and methods

Simulation was used to design a protocol and then to train over a two-week period all HCWs who might be involved in a SARS cardiac arrest.

### Phase 1: Cardiac arrest protocol

A modified ACLS protocol was designed and referred to as 'Code Blue Special' (CBS). We were aware that there was minimal scientific evidence, and there were no guidelines, for decisions related to having HCWs apply protective equipment that would delay time to definitive ACLS care. The Critical Care Department convened committee meetings involving experts representing the disciplines involved in the treatment of cardiac arrest (anesthesia, cardiology, critical care, emergency medicine, nursing, and respiratory therapy). The infection control service provided consultants to the committee. An initial protocol was developed by this committee.

**Figure 1:**
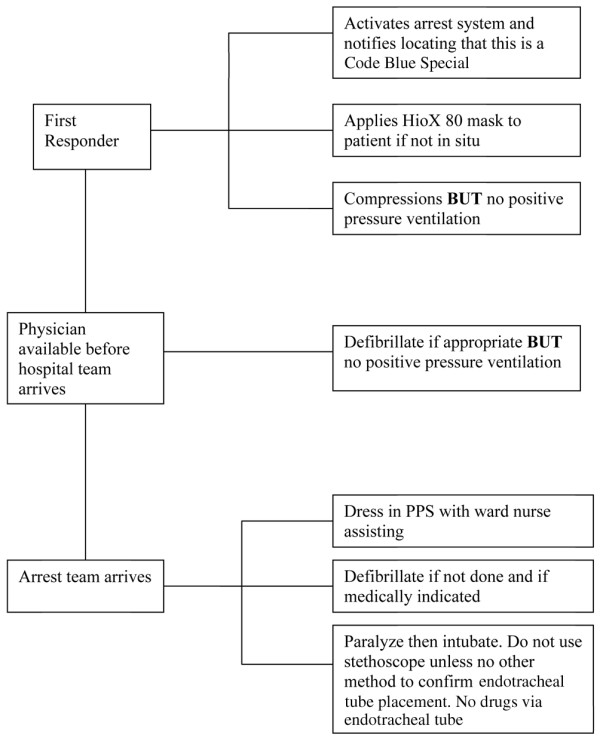
Summary of algorithm for cardiac arrest protocol (Code Blue Special) for a SARS patient.

A group of educators then assessed this protocol in a teaching area by repeated simulations. The infection control service monitored the simulations for breaches of infection control. After these simulations, discussions between educators and infection control personnel resulted in a modified protocol that was accepted by the multidisciplinary committee (Figure 1). During these simulations we recognized the need for a SARS-specific equipment cart.

Finally, the group of educators conducted a cardiac arrest simulation with a manikin (Laerdal™, SimMan) placed in a bed in an empty negative-pressure patient room on a ward. In preparation, all necessary equipment to manage a SARS cardiac arrest was placed outside the room and all HCWs that would respond to an actual SARS cardiac arrest (nurses, physicians and respiratory therapists) were present. A full arrest scenario was then simulated, including the transport of the resuscitated patient to the intensive care unit (ICU). During this simulation an educator and the director of infection control noted any flaws. Phase 1, the protocol development, took 4 days to complete.

### Phase 2: Team training program

The goal of Phase 2 was to train the on-call cardiac arrest teams in CBS. We acquired a dedicated training area in the hospital consisting of five adjoining rooms with computer and Internet access. We obtained call schedules for the arrest teams and began the training with the team members who were on call during the next two days.

Using our experience in Phase 1, we decided to train HCWs in groups of eight. We planned to train HCWs in the use of the PPS in groups of two, and because four educators were available daily we decided that the maximum number of HCWs for each session was eight.

**Figure 2:**
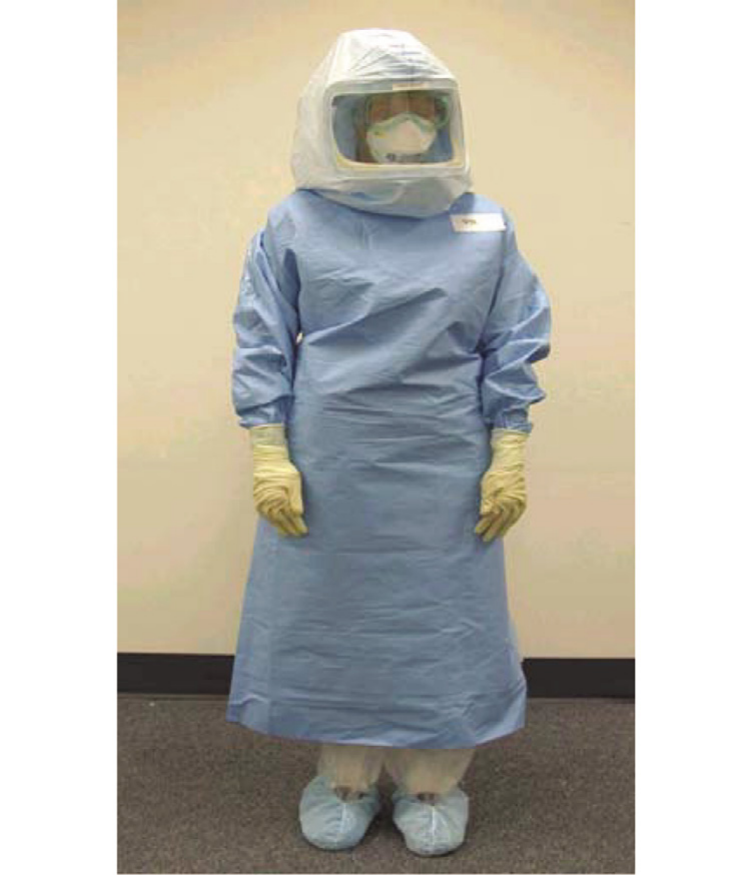
Healthcare worker dressed in T4 Stryker personal protection system (PPS). The PPS is worn over a disposable gown. In addition, goggles, an N95 respirator and two pairs of gloves are worn.

A two-hour training session proceeded as follows:

1. All HCWs attended a PowerPoint presentation highlighting pertinent principles for the care of the SARS cardiac arrest patient. Each received a handout and had time for questions and answers. We stressed all the modifications to the standard ACLS protocol, especially relating to defibrillation, airway management and infection control.

2. The group then observed two educators describing and demonstrating the dress-up and the dress-down method for the PPS.

3. Next, the group was divided into two sets of four who went into separate rooms. Here individualized practice sessions were done with the trainees donning and then removing the PPS (Figure 2). A 2:1 ratio of trainees to supervising educators was used.

4. Last, we simulated cardiac arrest scenarios. Four trainees managed one unknown ACLS scenario (asystole, pulseless electrical activity, pulseless ventricular tachycardia, and ventricular fibrillation). We timed how long it took the first person to don the PPS. The other four trainees observed.

5. After the simulation we debriefed the entire group and discussed and reinforced pertinent points. Then the four remaining HCWs managed a different simulation. The groups were chosen to mimic the arrest team, namely an anesthesia resident, ICU nurse, medical resident and respiratory therapist.

6. All physicians trained with their peers concurrently on call. There were six anesthesia residents and six medical residents, but many more nurses and respiratory therapists, to be trained. Hence, once we had trained the residents, we modified the simulation for the remaining nurses and respiratory therapists. Subsequent simulations involved only basic cardiopulmonary resuscitation and intubation because hospital policy mandates defibrillation by physicians only.

## Results

### Cardiac arrest protocol

#### Time to don the PPS

The first protocol required a PPS for everyone entering the patient's room as part of the arrest team. This was based on the assumption that it would take 1 1/2 minutes to don the PPS and this was felt to be an acceptable delay before providing patient care.

During Phase 1 we found that dressing took longer. When four members of the arrest team were simultaneously dressing it took between 3 1/2 and 5 1/2 minutes for the fastest team member to dress, even with assistants aiding verbally and physically. This longer time seemed to be due to HCWs and assistants talking at the same time to request equipment, and HCWs reaching across each other for equipment.

With the assistance of the logistics department we developed a cart for the PPS. The cart was easily portable and allowed four HCWs to access it simultaneously; it also had numbered equipment labels allowing HCWs to follow the dress-up procedure visually without memorizing the steps.

To expedite dressing we put up wall posters demonstrating the dress-up procedure and we used one dressing assistant per two team members. This represented a realistic number of people probably available to help at an actual arrest. It was also an acceptable total number of people (six) that could fit around the equipment cart.

#### Time to defibrillation

Once we discovered that the time to don the PPS in a team situation was at least 3 1/2 minutes, there was concern about the delay to defibrillation. After discussion with infection control and reviewing the available literature, we determined that there was no evidence that the person defibrillating needed to don a PPS. We therefore changed the protocol so that any physician on the ward could defibrillate, even if not part of the arrest team. This physician was required to wear routine protective SARS gear: an N95 respirator, goggles, a gown and two pairs of gloves. The N95 respirator is a face mask that filters 95% of particles greater than 0.3 μm in diameter. Respirator is the terminology used by the Centers for Disease Control and Prevention (USA) and the National Institute for Occupational Safety and Health.

We also proposed having all SARS patients on cardiac telemetry, so that a defibrillator could be brought into the room by the first responder. Available resources did not permit a defibrillator in each room.

#### Technique of defibrillation

Although a PPS was not worn for defibrillation, we noted that if instead of applying paddles, multifunction defibrillation electrodes capable of both pacing and defibrillation (M3501A; Agilent Technologies, Andover, MA, USA) were applied to the chest, hands-off defibrillation could be accomplished. The defibrillator machine could be placed about 2 m from the patient when multifunction electrodes were used, and the buttons on the machine could be pressed for defibrillation. There was uncertainty about the mode of transmission of the SARS virus, but 1 m is approximately the distance that organisms travel by droplet spread [14]. We recommended that, on wards with SARS patients, all defibrillators have this multifunction capability.

#### Ergonomic factors

A problem we encountered previously with the PPS was the risk of dislodging the PPS helmet when a stethoscope was placed in the ears [5]. To minimize stethoscope use, we used a portable end-tidal CO_2 _detector as the initial method to confirm tracheobronchial placement of the endotracheal tube. If the end-tidal CO_2 _was felt to be unreliable owing to low cardiac output we allowed a second person to place the stethoscope earpieces under direct vision. We did not use an esophageal detector device because of infection concerns with applying negative pressure to the airway of a SARS patient.

We also noted ergonomic limitations when wearing the PPS such as the following: claustrophobia; an inability to balance when removing equipment, which increased the risk of self contamination; the need to perform shorter periods of cardiopulmonary resuscitation to avoid heat fatigue; and the need to have an easy-to-follow poster of degowning placed on the wall to avoid making errors during the degowning process.

#### ACLS modifications

Positive-pressure ventilation was permitted only by HCWs wearing the PPS. To minimize the exposure of HCWs to the SARS virus, patients received a neuromuscular blocker before intubation. *In situ *intravenous access was added to the protocol to expedite drug delivery. No drugs were permitted via the endotracheal tube.

#### Exiting the patient room

During Phase 1 we noted that once the arrest team dressed in the PPS entered the room and began resuscitation, the initial HCWs in the room without a PPS were at risk. HCWs without a PPS were therefore instructed to position themselves at least 2 m from the patient during positive-pressure airway manipulation.

#### Infection control skills

During the simulations it became apparent that many seemingly simple actions during removal of the PPS were more complicated than expected and had been inadequately described. An example was the difficulty in removing the contaminated outer pair of gloves without contaminating the clean inner pair of gloves. Most instructions merely instruct one to 'remove the gloves'. The education and infection control team simulated every step of the dressing and undressing to ensure safety and clarity, and then scripted and photographed the process.

#### Composition of SARS cardiac arrest team and the number of HCWs to train

The usual cardiac arrest team at the time had ward nurses assisting the arrest team. This was changed during Phase 1 because it would have required training too many ward nurses in the application of the required PPS. Instead, ward nurses were trained as dressing assistants.

#### Negative pressure rooms

When the cardiac arrest was simulated in a negative-pressure room on the ward, we noted that there were items in the room that could not be disinfected, such as cork bulletin boards. Subsequently, we went to every negative-pressure room in the hospital to ensure infection control safety.

#### Lack of transport policy

During the planning for the simulation on the ward, we realized that the CBS protocol lacked a scripted transport policy for moving the resuscitated patient from the ward to the ICU. This policy was immediately developed in conjunction with the infection control, housekeeping and security services.

### Team training program

We trained 275 HCWs over a two-week period. Training sessions were held on Monday to Friday. All physicians were successfully trained in teams that mirrored their on-call schedule. The largest group to train was the 225 ICU nurses. It was difficult to free eight nurses for two hours because of concurrent patient care obligations and because we could not run sessions during ICU breaks. We ran three sessions during the day shift (07:30 to 19:30) and one session during the evening shift (20:00 to 22:00).

### Evaluation

The program was evaluated by the trainees. They were asked to complete an evaluation form at the end of each session. The results are summarized in Table 1. These forms were reviewed by the educators daily to review any concerns raised by the trainees.

During each session the educators checked both individual and team performances of the trainees in the cardiac arrest protocol, as well as the infection control policy.

We noted and corrected common ACLS deficiencies, for example an inability to attach the multifunction pacing/defibrillation electrodes to the defibrillator machine, or an inability to adjust the transcutaneous pacemaker settings such as the pacemaker output.

Mistakes in infection control practice by HCWs were noted and corrected. Common errors noted were the inability to remove the contaminated outer pair of gloves without contaminating the clean inner pair of gloves, the inability to remove the gown without contaminating the uniform underneath, the failure to disinfect hands appropriately, and not administering neuromuscular blocking agents before intubation.

The theme that needed constant attention was that removing the PPS always posed a great danger of self-contamination. Trainees were required to repeat the PPS removal until no errors in technique were noted. There were no known instances of self-contamination of HCWs in our institution. The effect on bedside practice was difficult to evaluate properly because only one cardiac arrest actually occurred in a patient suspected of having SARS.

## Discussion

We describe the use of high-fidelity simulation to design a modified practice of cardiac arrest resuscitation for an 'at risk of contamination' situation and to train caregivers as individuals and as a team. Simulation was used to delineate flaws and omissions in a modified ACLS protocol. We used scenario-based simulation training as an educational tool for different cardiac arrest etiologies. In all, 275 HCWs were trained in this SARS-specific cardiac arrest protocol.

One unexpected but crucial result was that the time to don the PPS was prolonged for a group (3 1/2 to 5 1/2 minutes) in comparison with a single HCW (1 1/2 minutes) donning the same equipment. This observation resulted in a major change to the initial protocol, namely not requiring the wearing of a PPS for defibrillation. A PPS was mandatory for any positive-pressure airway manipulation. We designed the protocol to minimize HCW contact with airway secretions.

We were concerned with the possibility of human error in this scenario, especially because of the reported transmission of SARS to protected HCWs involved in the intubation of a SARS patient [3].

We had to repeatedly reinforce our observation that although applying the PPS correctly was important, it was the undressing and removal of contaminated clothing that was even more important to prevent self-contamination. Undressing had to be done without the use of the dressing assistant, while wearing multiple layers of protective equipment. When simulation occurred in a negative-pressure patient room instead of the teaching area, we discovered unexpected infection control problems with furniture as well as our lack of a scripted transport protocol to move the patient to the ICU.

Some limitations of our approach became apparent and may help in planning for future disasters. Specific logistical challenges noted during our training period included the following:

1. The need for educators to have dedicated time freed from their regular duties.

2. The need for a high ratio of educators to trainees, to ensure careful observation of newly learned infection control practices.

3. The need for night training sessions for staff who work only night shifts.

4. The limited time in a crisis situation to simulate multiple scenarios.

5. The ongoing need for resources such as a dedicated training area, supplies and assistants.

6. The difficulty of quickly freeing up HCWs to train when they also have patient care obligations.

Because of the urgent nature of the crisis and time restraints we were unable to make a full evaluation of the effectiveness of our training. We evaluated only satisfaction with the program content, namely level one of the four levels of evaluation according to Kirkpatrick's model [15]. Although we considered evaluations before and after teaching, the limited time available to HCWs to attend the teaching sessions precluded this. We cannot validate the efficacy of our teaching because only one cardiac arrest occurred in the hospital in a patient suspected to have SARS.

The cost of the training program was substantial, although we do not have exact totals. This would include time for the dedicated educators, costs for educational materials and costs of the non-reusable equipment. In addition we needed two assistants. One was responsible for bookings, providing handouts, keeping sign-in records and collating evaluations. A second assistant was required to restock disposable equipment (for example gloves, gowns and masks) and to clean the rooms between sessions. Finally this project monopolized the high-fidelity simulator, excluding its use by others.

Planning can improve crisis management for future disasters. High-fidelity simulations of infectious disease protocols can be an invaluable asset for staff and patient safety. A written protocol can be developed and simulated, and core groups of people can be trained in the protocol before the crisis occurs. Once the crisis occurs, some HCWs should be immediately transferred from their usual duties to manage the patients. The other previously trained HCWs would immediately begin arranging training sessions in a pre-identified training area.

## Conclusion

High-fidelity simulation proved to be a crucial tool in the evaluation and implementation of a new, urgently developed SARS-specific cardiac arrest protocol, as well as in the subsequent training of team members in the use of unfamiliar protective equipment. It was used to detect and correct flaws and omissions in a theoretical protocol specific to the SARS patient. We used scenario-based simulation training to prepare our HCWs to manage a cardiac arrest in a SARS patient.

## Key messages

• 	We found that simulation was a valuable tool for evaluating a new treatment protocol in a novel and rapidly evolving crisis.

• 	We found that scenario-based simulation training was effective, but resource intense.

• 	We found that simulation was suited to teamwork training for disaster management.

• 	We suggest that simulation be used to prepare precise protocols for future serious events.

• 	We recommend that for uncommon events, simulation be done both in the teaching area and the actual patient environment.

## Abbreviations

ACLS = advanced cardiac life support; CBS = Code Blue Special; HCW = healthcare worker; ICU = intensive care unit; PPS = personal protection system; SARS = severe acute respiratory syndrome.

## Competing interests

The authors declare that they have no competing interests.

## Authors' contributions

SDA and SC were the lead educators in developing the cardiac arrest protocol and in arranging and delivering the simulation-based training. SDA was the lead writer of the article. SC reviewed the article. FB was involved in helping to develop the cardiac arrest protocol and was involved in the proof reading of the article. All authors read and approved the final manuscript.

## Figures and Tables

**Table 1:** Results of evaluation of training session for Code Blue Special

Question	Rating				
	
	1	2	3	4	5
1. Was the session comprehensive enough?			1	29	100
2. Was the duration of the session appropriate?			4	8	49
3. Were the teaching methods effective?				10	54

Question 1 was asked of all participants; questions 2 and 3 were added later. Scale: 1 = absolutely no, 5 = absolutely yes.
